# Hyperpolarized ^13^C Spectroscopic Evaluation of Oxidative Stress in a Rodent Model of Steatohepatitis

**DOI:** 10.1038/srep46014

**Published:** 2017-04-20

**Authors:** David M. Wilson, Valentina Di Gialleonardo, Zhen J. Wang, Valerie Carroll, Cornelius Von Morze, Andrew Taylor, Victor Sai, Mark VanCriekinge, Robert Bok, Michael A. Ohliger, Kayvan R. Keshari

**Affiliations:** 1 Department of Radiology and Biomedical Imaging University of California, San Francisco, San Francisco, CA 94158, USA; 2 Department of Radiology Memorial Sloan Kettering Cancer Center (MSKCC), New York, NY 10065, USA; 3 Molecular Pharmacology Program Memorial Sloan Kettering Cancer Center (MSKCC), New York, NY 10065, USA; 4 Weill Cornell Medical College, New York, NY 10065, USA

## Abstract

Nonalcoholic fatty liver disease (NAFLD) has become highly prevalent, now considered the most common liver disease in the western world. Approximately one-third of patients with NASH develop non-alchoholic steatohepatitis (NASH), histologically defined by lobular and portal inflammation, and accompanied by marked oxidative stress. Patients with NASH are at increased risk for cirrhosis and hepatocellular carcinoma, and diagnosis currently requires invasive biopsy. In animal models of NASH, particularly the methionine-choline deficient (MCD) model, profound changes are seen in redox enzymes and key intracellular antioxidants. To study antioxidant status in NASH non-invasively, we applied the redox probe hyperpolarized [1-^13^C] dehydroascorbic acid (HP DHA), which is reduced to Vitamin C (VitC) rapidly in the normal liver. In MCD mice, we observed a significant decrease in HP DHA to VitC conversion that accompanied hepatic fat deposition. When these animals were subsequently placed on a normal diet, resonance ratios reverted to those seen in control mice. These findings suggest that HP DHA, a potentially clinically translatable imaging agent, holds special promise in imaging NASH and other metabolic syndromes, to monitor disease progression and response to targeted therapies.

Nonalcoholic fatty liver disease (NAFLD) is now a highly prevalent disease in Western industrialized nations, often accompanied by other metabolic syndromes, namely obesity, insulin resistance, and hyperlipidemia^1^. A recent survey-based study found a 30% prevalence of NAFLD in the United States between 2011 and 2012^2^. The progression of simple steatosis to non-alcoholic steatohepatitis (NASH) disease shows histologic findings similar to that seen in alcoholic liver disease namely ballooning degeneration, and inflammation in hepatocytes^3^. This progression is of particular interest, since the latter is associated with cirrhosis and/or hepatocellular carcinoma (HCC), which may both be fatal. In NASH, triglyceride accumulation in the liver is accompanied by a significant inflammatory response, excess production of extracellular matrix, and oxidative stress^4^,^5^. Reactive oxygen species (ROS) can directly damage the cell via membrane lipid peroxidation, and exert redox-dependent metabolic alterations^6^. These metabolic changes are enforced by regulation of key enzymes, redox-dependent post-translational protein modifications, and control of nuclear receptors including the peroxisome proliferator-activated receptor (PPAR), proliferator-activated receptor-gamma coactivator-1 (PCG-1) and sterol response element binding protein (SREBP) families^7^,^8^,^9^.

Changes in redox status have been explored in numerous animal models of NASH, including those harboring genetic defects (*ob/ob*), or generated by feeding animals an altered diet^10^. The model that best recapitulates the histopathological features of NASH is the methionine-choline deficient (MCD) dietary model, which is accompanied by steatohepatitis, inflammation, and oxidative stress^11^,^12^. The dynamics of oxidative/nitrosative stress in the MCD model were recently studied at 2,4, and 6-week time-points showing reductions in liver catalase and superoxide dismutase (SOD) activities, and subtle but statistically significant elevations of total nitrates/nitrites (NO_x_)^13^. Both metabolic and proteomic analyses have been performed on MCD mice, yielding a large number of correlates to the observed oxidative injury in NASH^14^,^15^. The proteomic study conducted by Lee *et al*. by MALDI-TOF analysis showed that several of the differentially expressed proteins in MCD mice were related to redox homeostasis. These included proteins directly involved in the detoxification of reactive oxygen and reactive nitrogen species (glutathione peroxidase, superoxide dismutase and glutathione S-transferase), proteins functioning in cellular protection against oxidative stress (peroxiredoxins), and the microsomal enzyme cytochrome P450 2E1 (CYP2E1). The study showed a dramatic increase CYP2E1 in MCD-fed mice compared to normal diet, reinforcing its role in generation of reactive oxygen species^16^. Finally, some studies have shown a decreased concentration of reduced glutathione (GSH) in MCD-fed mice^13^,^17^,^18^, although this decrease was not observed at two weeks^13^.

Given the importance of oxidative stress in promoting hepatic injury in NASH, we studied the MCD-fed mice using a new spectroscopic method, namely magnetic resonance spectroscopy (MRS) using hyperpolarized [1-^13^C] dehydroascorbic acid (HP DHA), the oxidized form of Vitamin C (VitC) as an *in vivo* redox sensor^19^. Hyperpolarized ^13^C MRS is a relatively new technique by which the spin polarization of a nucleus is enhanced by several orders of magnitude (up to 10^5^) thereby allowing real-time study of *in vivo* metabolism^20^,^21^. Recently, the technique has been used in prostate cancer patients, demonstrating its potential for translation into routine clinical practice^22^. We developed HP DHA to study the changes in redox homeostasis that accompany cancer and other diseases (Fig. 1). HP DHA is transported rapidly into cells via glucose transporters (predominantly GLUT 1,3,4) and reduced to VitC in both cell and animal models^19^,^23^. This conversion is believed to occur in a GSH-dependent manner, catalyzed by number of enzymes including glutaredoxin, protein disulfide isomerase, and glutathione transferases^24^,^25^,^26^,^27^. We have also observed decreased HP DHA to VitC conversion in a model of diabetic nephropathy using *db/db* mice, and correlated this finding both to decreased GSH and increased NADPH oxidase 4 (Nox4) expression, reflecting increased superoxide generation^28^. For these studies, the rate of HP DHA to VitC conversion is best characterized by the resonance ratios derived from *in vivo*^13^C spectra, for example VitC/DHA or VitC/VitC + DHA. This conversion occurs within seconds in many tissues, reflecting rapid reduction of HP DHA by the cellular antioxidant network.

In the present study, we investigated NASH in the MCD-diet murine model using HP DHA and correlated spectroscopic data with hepatic steatosis. Fat accumulation in the liver was demonstrated both histologically and using ^1^H MRI fat-water imaging at ultra high-field (14 T). MCD-diet mice were also studied following return to a normal diet (MCDr or “recovery” group). Rapid imaging using HP DHA in a rodent model of NASH provided a means of demonstrating oxidative stress non-invasively and the showing the restoration of liver cell redox capacity in MCDr mice.

## Results

### MCD-fed mice showed significant lipid accumulation at two weeks as evaluated by ^1^H MRI at 14 T and histologic staining

**Figure 2:**
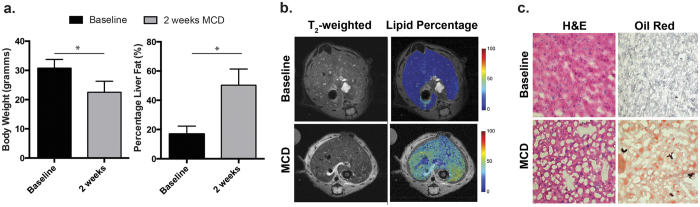
Lipid content of the liver in MCD-diet mice investigated by ^1^H MRI and histologic analysis. (**a**) Consistent with prior reports, a 27% decrease in total body weight was observed for MCD mice (**b**) Fat-water ^1^H imaging revealed a 3-fold increase in hepatic fat content. (**c**) Representative ^1^H MRI images from normal and MCD-diet mice by standard T_2_-weighted and fat-water imaging. Studies were conducted on a vertical wide-bore 14T magnet. While only subtle changes were observed using the T_2_-weighted sequence, dramatic hepatic steatosis was seen using the fat-water method. (**d**) Representative histologic sections using both hematoxylin and eosin as well as Oil Red O staining. The former shows microvesicular steatosis, while the latter shows significant lipid staining.

Animals fed the MCD-diet were characterized by decreased body weight and liver size at two weeks, consistent with prior reports (Fig. 2A). In the MCD group, the average hepatic volume was 0.57 ± 0.15 cm^3^ (0.69 ± 0.17 cm^3^ in the control group) and the average weight was 22.5 ± 3.8 g (30.8 ± 2.9 g in the control group), representing a 27% decrease (p < 0.05). The presence of oxidative injury in MCD mice was demonstrated *via* a catalase activity assay, and anti 8-OHdG monoclonal antibody staining (Figure Supp Fig. 1). These studies were consistent with prior reports showing decreased catalase activity and increased 8-OHdG in MCD mice^13^,^29^. Fat content of the liver was investigated by ^1^H MRI, routine histology, and specialized staining. Steatosis was first demonstrated *in vivo* using ^1^H MRI fat-water technique^30^, showing a significant increase in hepatic fat content (Fig. 2B, C). In the MCD group, the hepatic fat content was 50.3 ± 11.1%, versus 16.9 ± 5.4% in the control group corresponding to a 3-fold increase (p < 0.05). In contrast, conventional T_2_-weighted sequences showed minimal differences in parenchymal signal between MCD and control groups. Following sacrifice, paraffin-embedded sections were studied using hematoxyln and eosin staining, showing predominantly microvesicular steatosis, without evidence of fibrosis (Fig. 2D). This appearance is consistent with the study published by Jorgacevic *et al*., which explicitly studied the dynamics of histologic changes in MCD-fed animals^13^. The liver was also studied using Oil Red O dye, a lysochrome diazo dye used to stain triglycerides and lipids. Marked accumulation of the stain was observed for MCD-derived liver samples.

### Hyperpolarized ^13^C dehydroascorbate spectroscopy reveals decreased HP DHA to VitC conversion in MCD-fed mice relative to controls

The redox sensor HP DHA was used to evaluate altered redox capacity in MCD-diet mice, using a ratiometric, 3D spectroscopic technique. In this case a single time-point ^13^C magnetic resonance spectroscopic imaging (MRSI) study was performed to detect HP DHA to VitC conversion. Following intravenous injection of HP DHA, rapid conversion to VitC occurs in a matter of seconds, particularly in tissues with high exposure to oxidative stress including the brain, kidneys and liver. The rate of HP DHA to VitC conversion has been previously correlated with components of the redox network in animal models of cancer and diabetes^19^,^23^,^28^. As discussed previously, there is severe disruption of redox homeostasis in the MCD-deficient model, as evidenced by the downregulation of numerous enzymes involved in detoxification of reactive oxygen and reactive nitrogen species. We speculated that these numerous alterations might be detected by our non-invasive and potentially translatable HP MRS method.

**Figure 3:**
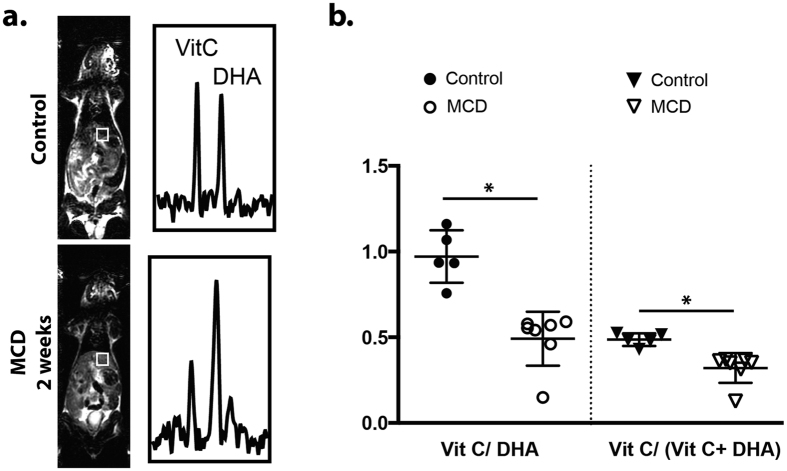
Study of control and MCD groups by HP DHA ^13^C MRSI. (**a**) Representative ^13^C spectra obtained from the livers of MCD-diet mice using HP DHA at two weeks. Voxels corresponding to the liver for both C and MCD mice are juxtaposed with T_2_-weighted images acquired at the time of the HP experiment. Resonances corresponding to [1-^13^C] DHA and [1-^13^C] VitC are depicted, with a clear decrease in the HP VitC to DHA ratio observed in the MCD-diet animals. (**b**) Scatter plots showing the metabolite ratios derived from animal experiments for the normal and MCD groups, expressed both as VitC/VitC + DHA and VitC/DHA. These ratios decreased significantly by 35% and 49% respectively (p < 0.05 for both) in the livers of MCD-diet animals relative to controls.

In control mice, intravenous administration of HP DHA followed by ^13^C MRSI revealed similar VitC/VitC + DHA and VitC/DHA metabolite ratios to those previously reported. In contrast, these ratios were markedly lower in MCD mice as shown in Fig. 3. The average VitC/VitC + DHA peak ratio was 0.49 ± 0.02 for the control group and 0.32 ± 0.03 for the MCD group (35% decrease, p < 0.05), while the average VitC/DHA metabolite ratio was 0.97 ± 0.07 for the control group and 0.49 ± 0.06 for the MCD group (49% decrease, p < 0.05) In all cases, anatomic T_2_-weighted ^1^H imaging was performed during the same session to confidently assign voxels to the hepatic parenchyma.

### MCD recovery mice showed a complete return to baseline by HP DHA MRS despite persistent elevated hepatic lipids

**Figure 4:**
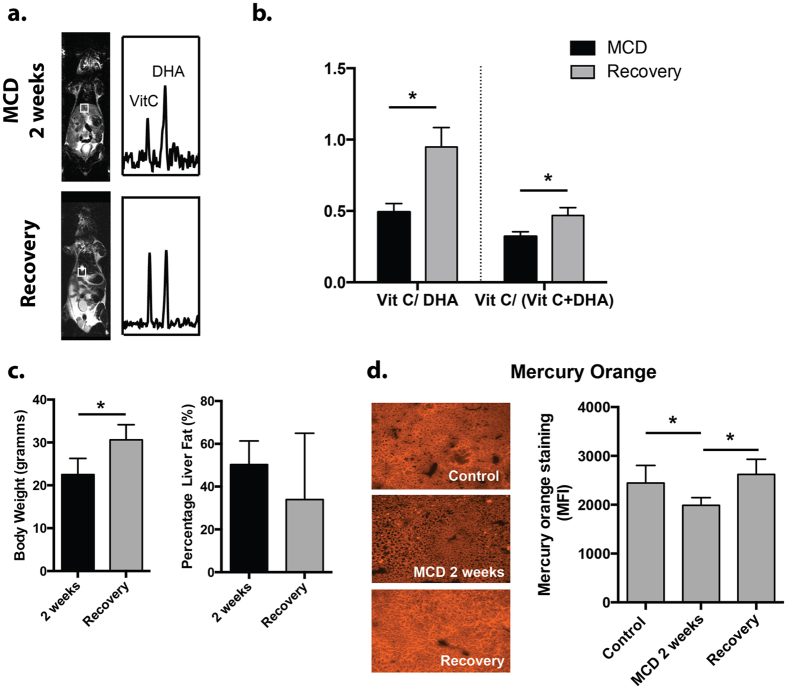
Study of recovery phase animals (MCDr group) by HP DHA. (**a**) Representative ^13^C spectra obtained from the MCD group are compared to those obtained from mice first fed the MCD diet, then normal chow for 1 week. The HP DHA to VitC resonance ratios in the recovery group appear similar to those obtained for controls. (**b**) Bar graphs obtained for MCDr mice show significant increases in VitC/VitC + DHA and VitC/DHA ratios (p < 0.05) following return to a normal diet, which were not significantly different from baseline mice. (**c**) Although body weights in MCDr mice returned to normal, there was still significant hepatic steatosis (30%) seen in these animals, 2-fold that seen in the control group. (**d**) Mercury Orange staining showing decreased thiol content in MCD mice. Significant normalization was seen in the recovery group.

**Figure 5:**
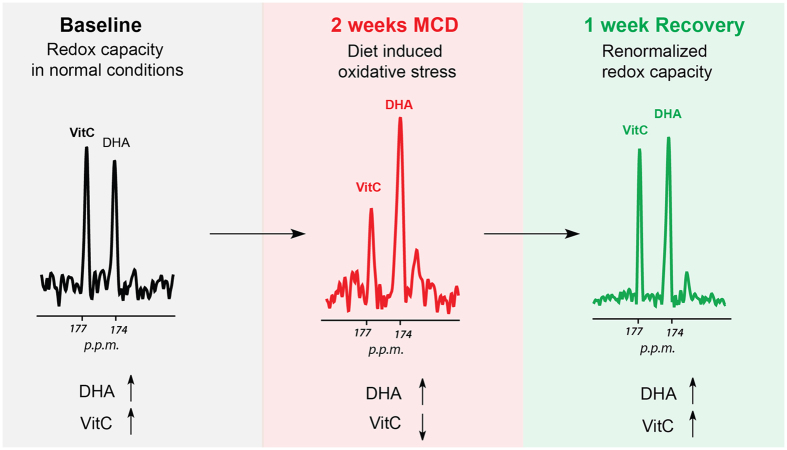
Model of oxidative stress imaging using HP DHA in the setting of NASH. In MCD mice, markedly decreased HP DHA to VitC conversion was seen compared to controls. The ^13^C spectra returned to baseline following return to a normal diet, while hepatic fat content remained significantly elevated.

Having shown that the redox sensor HP DHA is responsive to hepatic injury in the MCD-diet model, we studied recovered mice following return to a normal diet (MCDr group). Numerous other modulations of the redox network in MCD mice have been studied, using artificial enzymes, vitamins, and other natural products. These therapies have included Mn-salen complexes, Vitamin C, Chunggan extract, hydrogen sulfide, hypotaurine and curcurmin^14^,^15^,^31^,^32^,^33^. In our study, mice originally fed the MCD-diet were allowed to recover for one week on a normal diet (n = 4, MCDr group). The animals were imaged by ^13^C MRSI following injection of HP DHA with VitC/VitC + DHA and VitC/DHA resonance ratios again calculated for hepatic voxels. The results are shown in Fig. 4, where representative HP spectra for the MCDr group are shown, and the metabolite ratios derived are compared to those for the MCD group. These data showed nearly identical metabolite ratios to those calculated for the control group, with the average VitC/VitC + DHA 0.47 ± 0.05 and the average VitC/DHA 0.95 ± 0.13 (p > 0.05 versus C group). Sacrificed MCDr mice showed mild histologic improvement in hepatic steatosis versus the MCD group, but significantly elevated lipid content relative to control animals (Fig. 4C). Fat-water ^1^H imaging of MCDr animals showed an average fat content of 34.0 ± 31.0%, representing a 2-fold increase versus the control group (p < 0.05), but no significant change versus MCD animals. In addition, the presence of thiols was assessed using the Mercury Orange technique showing depletion in MCD animals and recovery in MCDr mice (Fig. 4D)^34^. These key findings lead to a model of MCD induced oxidative stress (Fig. 5).

## Discussion

This study demonstrated that ^13^C MR spectroscopy using HP DHA is a sensitive indicator of metabolic alterations in the MCD-diet model of NASH. Given the extensive data implicating oxidative stress in this model, as well as what is known about the mechanism of HP DHA to VitC conversion, it is likely that altered redox status in MCD-mice is responsible for the observed differences in metabolite ratios. Oxidative stress in MCD-mice occurs via numerous mechanisms including decreased concentrations of intracellular reducing agents, decreased expression of key antioxidant defense enzymes (GPx, SOD, GST, and members of Prx family), and enhanced activity of microsomal enzymes especially CYP2E1. An interesting finding of our study was decreased overall thiol staining in the livers of MCD mice versus controls, in the absence of significant differences in GSH. This result may reflect the oxidation of protein thiols (for example thioredoxins), which some studies suggest represent the largest cellular redox pool^35^.

Given the complexity of the intracellular redox network, it is difficult to identify which components are primarily responsible for *in vivo* HP DHA to VitC reduction. HP DHA has been previously studied in lymphoma (EL4) cells^23^, the transgenic adenocarcinoma of the mouse prostate (TRAMP) model^36^, and the *db/db* murine model of diabetic nephropathy^28^. These studies also analyzed specific components of the redox network and found correlations with the observed *in vivo* behavior of HP DHA. Given the results of these studies as well as what is known about DHA metabolism, HP DHA is probably best considered an oxidative challenge to tissues, with altered conversion seen in conditions for which the antioxidant defense system is compromised. Specific modulations of the redox network, *via* transgenic mice and gene silencing in cell culture, are currently being used to further elucidate the *in vivo* mechanisms of HP DHA and other novel redox probes. In addition, technical aspects of the HP DHA-MRI experiment are being refined, in particular to address toxicity *in vivo*^37^. Specifically, polarization and MR acquisition methods will require improvement, to allow the ^13^C DHA dose reduction needed for patient studies.

In summary, HP DHA in a NASH model allowed us to assess the oxidative damage associated with disease progression and its normalization upon return to a normal diet. Moreover, in recovering mice this effect was reversed before the resolution of intrahepatic lipid deposits, suggesting that modulations of the antioxidant network and lipid accumulation may be non-synchronous in NASH. This finding has great implications both for potential use of HP DHA in the clinic as well as the development of novel therapeutic strategies to address NASH.

## Methods

### Animals and experimental design

All procedures were approved by the Institutional Animal Care and Use Committee. Veterinary services for the study were provided by the UCSF Laboratory Animal Resource Center (LARC) and all studies were performed in accordance with UCSF guidelines regarding animal housing, pain management, and euthanasia. The experiments used male FVB/NJ mice with an initial weight of 25–35 grams. The animals were kept under standard laboratory conditions (temperature 22 ± 2**°**C, relative humidity 50 ± 10%, 12 h/12 h light-dark cycle). All animals were fed with a control diet for at least 1 week (acclimatization period). 3 groups of animals were studied; Group 1: control group (C; n = 5); group 2: MCD-diet group fed for 2 weeks (MCD; n = 7); group 3: MCD-diet group fed for 2 weeks, followed by 1 week return to normal diet (MCDr; n = 4). Standard diet contained 2 g/kg of choline chloride and 3 g/kg of methionine, while the MCD diet had identical composition as the normal chow but without methionine and choline (Dyets Inc., Supp Fig. 2). Animals in each group were subjected to HP DHA ^13^C MRS, ^1^H fat-water imaging, and histologic evaluation following sacrifice.

### Synthesis, preparation and polarization of [1-^13^C] dehydroascorbic acid

[1-^13^C] DHA (Isotec, Miamisburg, OH) was synthesized using a published method, by air oxidation of [1-^13^C] ascorbic acid in the presence of catalytic amounts of copper (II) acetate^38^. A 2.2 M solution of [1-^13^C] DHA in dimethyacetamide (DMA) containing 15 mM OX063 trityl radical (Oxford Instruments) was hyperpolarized on a HyperSense DNP instrument (Oxford Instruments) as previously described^19^. The frozen sample was dissolved in distilled water containing 0.3 mM EDTA.

### Hyperpolarized ^13^C magnetic resonance spectroscopic imaging

*In vivo* studies were performed using a 3T MRI scanner (GE Healthcare) equipped with the MNS (multinuclear spectroscopy) hardware package. The RF coil used in these experiments was a dual-tuned ^1^H-^13^C coil with a quadrature ^13^C channel and linear ^1^H channel construction as reported previously^39^. Prior to ^13^C studies, 3-plane T_2_-weighted images were acquired for anatomic localization (T_E_ = 100 ms, T_R_ = 4 s, 6 averages) using a standard fast spin echo sequence. These images were used to calculate liver volumes for the control, MCD, and MCDr groups. Single time-point ^13^C MRSI studies were carried out as previously published^39^. 350 μL of a hyperpolarized 15 mM [1-^13^C] DHA solution were injected similar to previously described methods for [1-^13^C] pyruvate. An 8 M ^13^C urea phantom in a sealed tube was placed within the sensitive volume of the RF coil. For the ^13^C 3D MRSI studies, the imaging sequence was initiated 10 s following completion of a 15 s injection (representing a total delay of 225 ms). ^13^C 3D MRSI used a variable flip angle scheme, matrix size of 8 × 8 × 16 (voxel size of 5 mm isotropic), TR = 105 ms, spectral bandwidth = 25000 Hz, 2538 points.

### Fat-water imaging at 14T

For all groups (control, MCD and MCDr), high-resolution T_2_ and fat-water imaging was conducted at 14T using a Varian WB600 micro-imager and 40 mm ^1^H millipede coil (Varian Instruments, Palo Alto, CA). Lipid percentage maps were calculated from fat and water images acquired with a conventional spin-echo sequence using the Dixon method^30^,^40^.

### Histopathology/Activity assays

Liver tissues from the control, MCD and MCDr groups were studied using Hematoxylin and Eosin (H&E), Oil Red O, and Mercury Orange staining. Formalin-fixed, paraffin-embedded liver tissues were sectioned at 4 μm and stained with Hematoxylin and Eosin; frozen tissues embedded in OCT (optimum cutting media) were sectioned at 10 μm and stained for Oil Red O and Mercury Orange using standard protocols^34^,^41^. Anti 8-OHdG staining was performed as reported previously^28^. Catalase activity was determined via a standard method on homogenized liver tissue^13^.

### Data analysis and statistical considerations

*In vivo* MRSI data were processed using custom software written in IDL 8 (ITT Visual Information Solutions) and Matlab 2009b (MathWorks). DHA and Vitamin C resonances were integrated and peak heights were used to calculate relevant ratios. In order to assign voxels to the liver, 100% of a given voxel corresponded to the tissue of interest as validated by the corresponding T_2_-weighted image. Metabolite ratios were calculated for individual voxels using HP ^13^C DHA and Vitamin C resonances, using relative peak heights. Average metabolite ratios (VitC/VitC + DHA and VitC/DHA) for liver tissue in individual animals were then calculated, and these values averaged for control, MCD and MCDr groups. For all animals, the number of voxels (per animal) used to determine the VitC/VitC + DHA and VitC/DHA ratios in the liver ranged between 2–4. All statistics were calculated using JMP software (SAS Corporation, Cary, NC). Significance was reported using a two-sided Student’s t-test for all comparisons and a p-value < 0.05. All data are reported as mean ± standard error, with corresponding error bars depicted in the Figures.

## Additional Information

**How to cite this article:** Wilson, D. M. *et al*. Hyperpolarized ^13^C Spectroscopic Evaluation of Oxidative Stress in a Rodent Model of Steatohepatitis. *Sci. Rep.* **7**, 46014; doi: 10.1038/srep46014 (2017).

**Publisher's note:** Springer Nature remains neutral with regard to jurisdictional claims in published maps and institutional affiliations.

## Supplementary Material

Supplementary Information

## Figures and Tables

**Figure 1:**
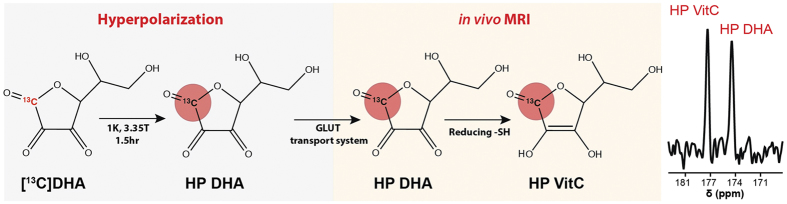
Imaging oxidative stress *in vivo* using HP [1-^13^C] DHA. The probe is polarized using the dynamic nuclear polarization (DNP) technique, in a concentrated solution containing an unpaired electron source. Following dissolution and intravenous injection, HP [1-^13^C] DHA is transported rapidly into cells via glucose (GLUT) transporters. Enzyme mediated two-electron reduction of [1-^13^C] DHA to [1-^13^C] VitC is detected spectroscopically. This conversion depends on cellular reducing capacity, which is diminished in the setting of oxidative stress.
