# Supplementary material for: Hyperpolarized 13C Spectroscopic Evaluation of Oxidative Stress in a Rodent Model of Steatohepatitis
Source: Sci Rep. 2017 Apr 20;7:46014. doi: 10.1038/srep46014 (PMC5397869; doi:10.1038/srep46014)
Supplement: Supplementary Information [file srep46014-s1.pdf]

Hyperpolarized ^{13}C Spectroscopic Evaluation of Oxidative Stress in a Rodent Model of Steatohepatitis

David M. Wilson[1], Valentina Di Gialleonardo[2,3], Zhen J. Wang[1], Valerie Carroll[1], Cornelius Von Morze[1], Andrew Taylor[1], Victor Sai[1], Mark VanCriekinge[1], Robert Bok[1], Michael A. Ohliger[1], Kayvan R. Keshari[2,3,4*]

Supplementary Information:

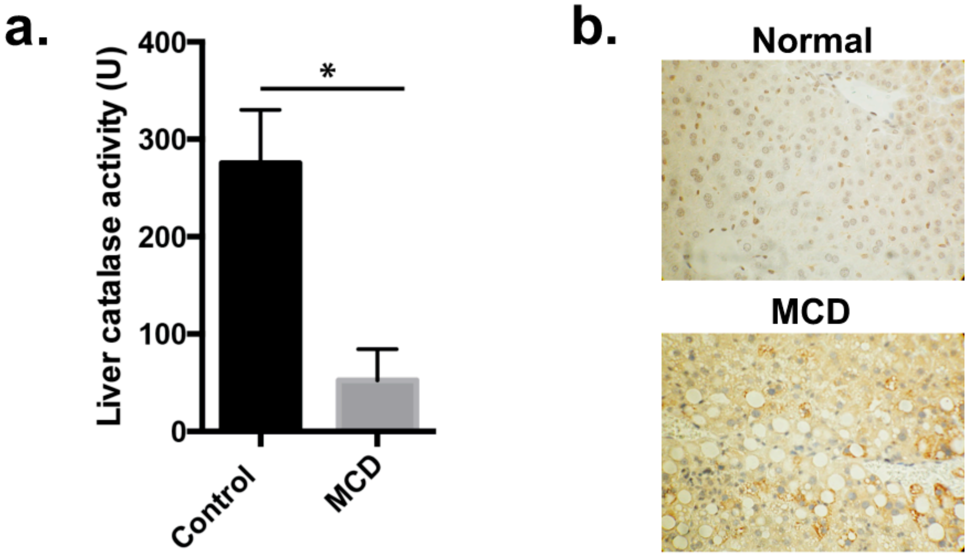

Supplementary Figure 1. Oxidative stress in the MCD model. (a) Catalase assay performed at two weeks shows reduced activity, consistent with prior reports. (b) Representative anti-8-OHdG immunohistochemistry, showing increased staining in the livers of MCD mice.

DYET# 518828

Custom L-AA Defined AIN-93G Diet without L-Methionine or Choline with Corn Oil as Fat Source

L-Arginine (free base)		*6.3*
L-Histidine (free base)		*4.5*
L-Lysine-HCl		*16.1*
L-Tyrosine		*9.2*
L-Tryptophan		*2.1*
L-Phenylalanine		*8.7*
L-Cystine		*3.7*
L-Threonine		*6.6*
L-Leucine		*15.3*
L-Isoleucine		*8.4*
L-Valine		*9.9*
Glycine		*3.1*
L-Proline		*20.4*
L-Glutamic Acid		*36.2*
L-Alanine		*4.5*
L-Aspartic Acid		*11.3*
L-Serine		*9.4*
	total L-AA*……….	175.7

Ingredient		gm/Kg
Cornstarch		150
Dyetrose		50
Sucrose		441.9
Cellulose		30
Corn Oil		100
Salt Mix #210030		35
Sodium Bicarbonate		7.4
Vitamin Mix #310025		10
	total ..	1000.00

Supplementary Figure 2. Detailed content of MCD diet employed in this study.
